# Performance of a Dedicated Radioprotection Cabin for the Interventional Echocardiographer During Structural Heart Procedures

**DOI:** 10.1016/j.shj.2025.100756

**Published:** 2025-11-10

**Authors:** François Magniez, Luis Ammour, Pierre-Guillaume Piriou, Nicolas Piriou, David Stevant, Quentin Bernard, Laurianne Le Gloan, Philippe Jaafar, Vincent Letocart, Julien Plessis, Thibaut Manigold, Robin Le Ruz, Pierre Yves Turgeon, Patrice Guerin

**Affiliations:** a Department of Cardiology, Institut du Thorax, Nantes University Hospital, Nantes, France; b Department of Cardiology, Institut Universitaire de Cardiologie et de Pneumologie de Québec (IUCPQ), Québec, Canada

**Keywords:** Cardiology, Echocardiography, Interventional, Occupational exposure, Radiation protection, Transesophageal

## Abstract

**Background:**

Structural heart procedures performed under fluoroscopy and transesophageal echocardiography guidance require the expertise of an interventional echocardiographer in the heart team. Due to the proximity with the radiation source, the physician might be exposed to high radiation doses. This study aims to assess the interventional echocardiographer radioprotection by means of a dedicated cabin and evaluate its ergonomic characteristics.

**Methods:**

This is an observational nonrandomized study comparing cumulative radiation exposure between a control arm, consisting of a standard lead glass panel, and the experimental arm using the dedicated radioprotective Echosafe cabin. All structural heart procedures requiring transesophageal echocardiography between July 2021 and April 2022 were included. Cumulative radiation exposure of the interventional echocardiographer was collected by the body part and the satisfaction of the heart team with the cabin was assessed with a questionnaire.

**Results:**

A total of 64 procedures were included (24 controls and 40 with the cabin). The irradiation (as estimated by dose area product per procedure) was similar between the two groups (*p* = 0.94). The use of the cabin resulted in a numerically lower cumulative radiation dose to each body part. The mean overall reduction in radiation dose was estimated to be between 56 and 78% with the cabin. The most frequent negative aspects reported by the cabin users included difficulty moving the transesophageal echocardiography probe and the physical burden associated with initial installation.

**Conclusions:**

This preliminary study suggests that radioprotection of the interventional echocardiographer can be increased during structural procedures by using a dedicated cabin. Further studies with larger sample sizes and different design will be needed to confirm the reduction in irradiation of the echocardiographer.

## Highlights

• The Echosafe cabin is a new device specifically designed to protect invasive echocardiographers from radiation during structural heart interventions.
• It provides a significant reduction in radiation exposure for each body part of the echocardiographer.
• It is practical and ergonomic in routine daily use.

## Graphical abstract

**Figure:**
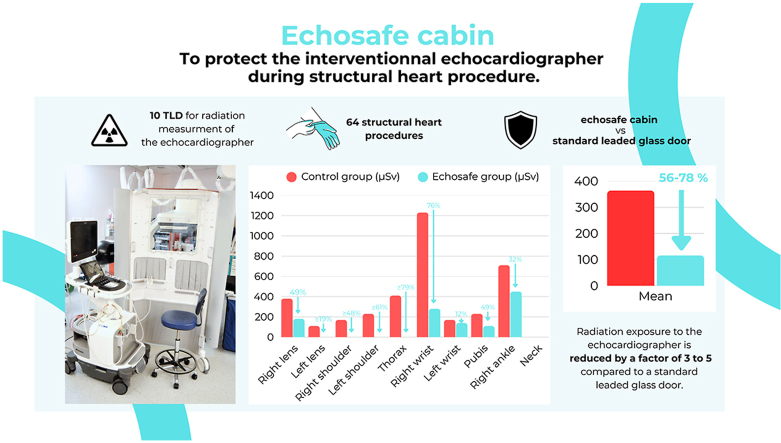

## Introduction

Structural cardiology has experienced remarkable growth since the early 21st century and has become a central pillar in the treatment of heart disease. However, the use of X-rays for fluoroscopic guidance during these procedures exposes medical workers to a significant radiation level^1^, ^2^, ^3^ and increases occupational health hazards, including radiation-induced cancers,^4^^,^^5^ carotid stenosis^6^ and cataracts.^7^^,^^8^ Given the increasing number of structural cardiology procedures performed, the echocardiographer, who guides the procedure using transesophageal echocardiography, plays a central role in the catheterization laboratory. Due to the proximity of the X-ray tube and the lack of a dedicated radioprotection device, exposure to radiation may be up to five times higher for the echocardiographer than for the interventional cardiologist.^9^

To protect the echocardiographer from radiation in the catheterization laboratory, the interventional cardiology team from the Nantes University Hospital (France) has developed, in close collaboration with the local company Lemerpax (La Chapelle sur Erdre, France), a radioprotective device dedicated to the echocardiographer, which has been called the Echosafe cabin. The aims of the study are to evaluate the efficacy of this cabin to protect the echocardiographer from radiation and to assess the ergonomic characteristics of the device.

## Methods

### Data Availability Statement

The authors declare that all supporting data are available in the article and online supplement.

### The Echosafe Cabin

**Figure 1:**
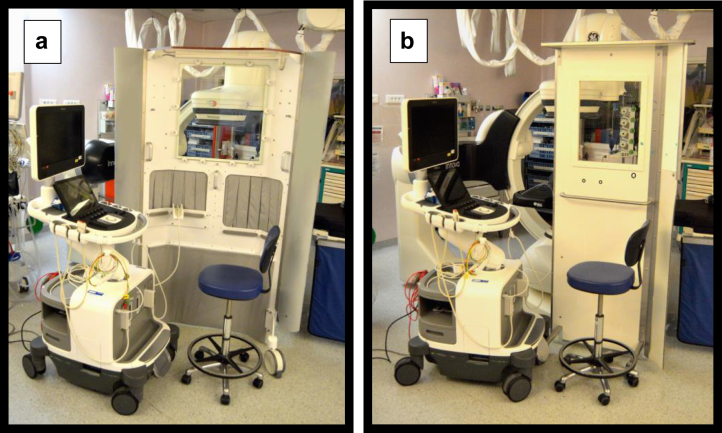
Photos of (a) the Echosafe cabin and (b) the control radiation protection device. (a) The Echosafe cabin allows introducing the transoesophageal probe through the left lead flap and the arm of the echocardiographer through the right flap. The X-ray tube is close to the patient’s head on the other side of the cabin, and the interventional cardiologists are located on the right of the cabin (radial and femoral approach). (b) The control radiation protection device, which was used previously in our hospital, is a lead glass door placed between the echocardiographer and the X-ray tube.

The Echosafe cabin was designed at the Nantes University Hospital and it was manufactured by the Lemerpax company. The cabin is equipped with a transparent screen, a protective ceiling, and 2 access points via lead flaps, 1 for the transesophageal probe and 1 for the echocardiographer's arm (Figure 1).

### Study Design and Procedures

This is a prospective, open-label, nonrandomized, single-center study conducted at the Nantes University Hospital (France) comparing radiation exposure with and without the Echosafe cabin. We included every structural cardiology procedure requiring transesophageal echocardiography between July 2021 and April 2022.

Patient inclusion in either study arm was left to the operator’s discretion. Procedures were sequentially assigned to either the echosafe or control group over the course of the study, with the allocation designed to maintain comparable levels of total irradiation between groups, as estimated by the dose area product (DAP).

In the Echosafe group, the procedures were performed using the Echosafe cabin, whereas in the control group, a lead glass panel was placed between the echocardiographer and the X-ray tube as routinely performed previously in our institution (Figure 1). The echocardiographers and other workers from the catheterization laboratory were trained on cabin use (placement of the echocardiographer and the echocardiograph, use of fluoroscopic X-ray equipment, and set up of sterile drapes).

For each procedure, patient morphological characteristics (age, sex, weight, and body mass index) and radiation exposure (DAP, air kerma (AK), and estimated patient effective dose) were recorded. For each dosimetry assessment, the distribution between fluoroscopy and radiography was collected. All procedures were performed in the same operating room, using the same fluoroscopy system (Monoplan GE Healthcare Innova IGS 520, Nantes, France).

### Radiation Measurement

**Figure 2:**
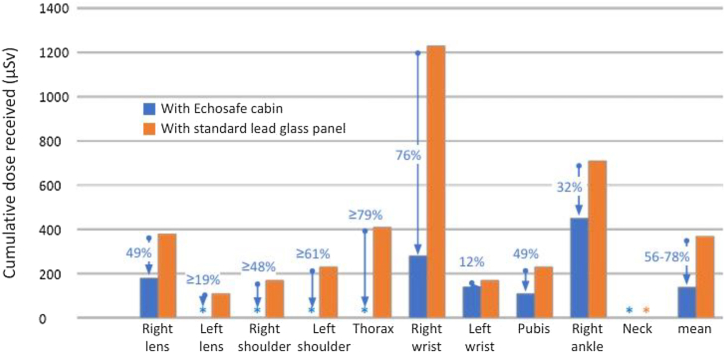
Cumulative dose received by the operator during the procedures performed with (n = 40) and without (n = 24) the Echosafe cabin (for each body part and mean). ∗Measurements below the 100 μSv detection limit radiation reduction are calculated after adjusting by a factor of 1.074 to account for the difference in dose area product between the two groups.

The echocardiographer radiation exposure was measured by 10 thermoluminescent dosimeters (TLDs), assigned to each study group. All TLDs were worn above personal protective equipment. Five Hp(0.07) TLDs were placed on the vest (1 on each shoulder, 1 on the left thorax, 1 on the pubis, and 1 at the back of the neck), 2 Hp(0.3) TLDs were on the branches of the glasses to provide an estimated dose to the lens, and 3 Hp(0.07) TLDs were set onto 3 bracelets (left and right wrists and right ankle). A control TLD was located in an area that was not exposed to radiation and it was used as a reference to account for natural radiation. The TLD lower limit of detection was 100 μSv. The cumulative dose received by the operator during the procedures (for each body part) is presented in Figure 2.

Radiation measurements were performed by the company that supplied the TLDs (Landauer, Vélizy-Villacoublay, France).

### Ergonomics Assessment

**Figure 3:**
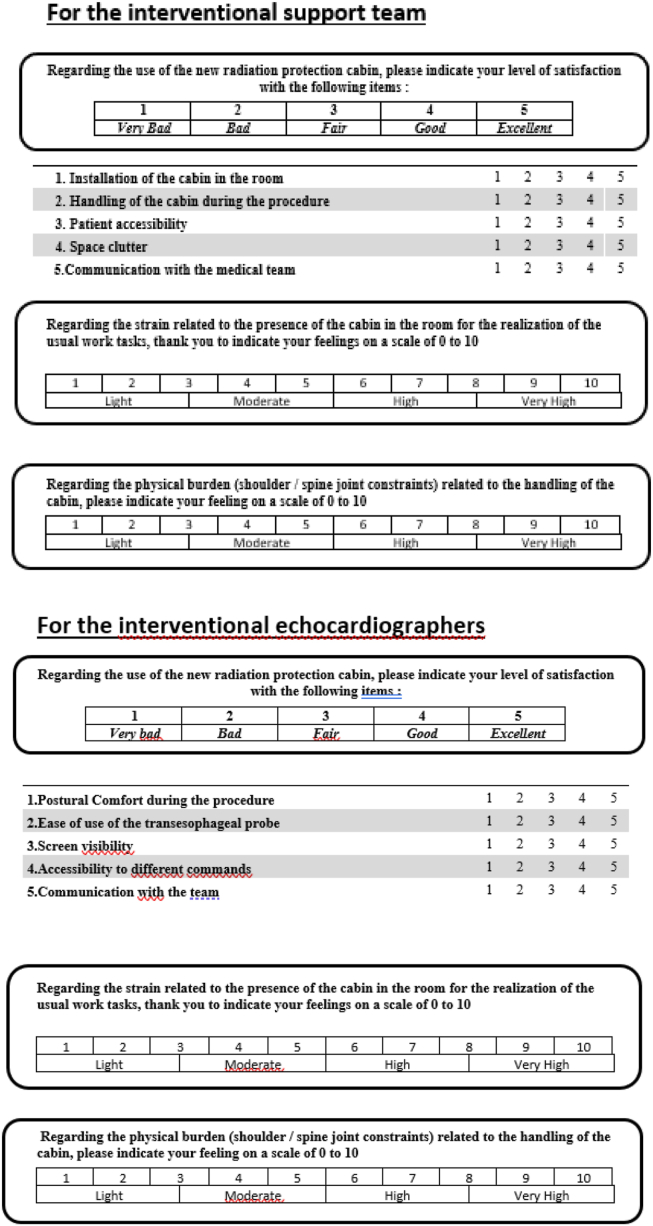
The satisfaction questionnaire.

To assess the Echosafe cabin ergonomics, we developed, with the assistance of an occupational physician from our hospital, a questionnaire dedicated to both the echocardiographers who use the device and the interventional support team members who install it and work around it (Figure 3). For each group, satisfaction was evaluated with 5 questions, rated from 1 (very bad) to 5 (excellent). The second part of the questionnaire focused on the strain and physical burden associated with the use of the cabin, assessed on a scale ranging from 1 (very light) to 10 (very high). The workflow of the invasive cardiologists or the anesthesiology team was clearly not disturbed by the device and was not formally evaluated.

### Statistical Analysis

Statistical analysis was performed using Fisher's exact test for categorical variables and Student’s t-test or Wilcoxon–Mann–Whitney test for continuous variables, depending on data distribution.

To calculate the decrease in cumulative radiation dose observed with the Echosafe cabin compared to control, the cumulative dose in the Echosafe group was first adjusted by a factor corresponding to the ratio of total DAP for the control group to total DAP for the Echosafe group.

### Ethical Approval

The study was approved by the local ethics committee “Groupe Nantais d’Ethique dans le Domaine de la Santé”.

## Results

Inclusions started in July 2021 and ended in April 2022. Forty procedures performed with the Echosafe cabin were included in the Echosafe group, and 24 procedures were performed in the control group (Table 1).

**Table 1:** Characteristics of the procedures and population

	Echosafe group n = 40	Control group n = 24	*p* values
Procedures			
Transcatheter edge-to-edge repair of the mitral valve	19 (47.5%)	13 (54.2%)	0.80
Atrial septal defect closure	14 (35.0%)	9 (37.5%)	>0.99
Paravalvular leak closure	2 (5.0%)	2 (8.3%)	0.63
Other	5 (12.5%)∗	0 (0%)	0.148
Procedure characteristics			
Total DAP (mGy cm^2^)	70.042	75.207	
DAP per procedure (mGy cm^2^)†	1007 [336-2311]	1067 [196-2734]	0.94
Total air kerma (mGy)	5880	7010	
Air kerma per procedure (mGy)†	79 [25-233]	77 [13-247]	0.90
Estimated patient effective dose (mSv)	196	249	
Estimated patient effective dose per procedure (mSv)†	2.82 [0.94-6.47]	2.99 [0.55-7.66]	0.91
Fluoroscopy time per procedure (min)†	15.7 [4.2-27.0]	15.9 [4.6-30.5]	0.55
Patients characteristics‡			
Mean age (years)	63.2 ± 19.8	66.4 ± 24.1	0.54
Gender (men)	23 (57%)	11 (46%)	0.44
Body mass index (kg/m^2^)	25.0 ± 4.7	24.85 ± 4.0	0.93

Abbreviation: DAP, dose area product.

∗ Including 1 mitral valvuloplasty, 1 transcatheter edge-to-edge repair of the tricuspid valve, 1 atriopulmonary fistula closure, 1 percutaneous mitral bioprosthesis implantation, and 1 percutaneous tricuspid bioprosthesis implantation.

† Values are median [interquartile range].

‡ Values are numbers (percentages) or means ± SDs.

The characteristics of the patients were similar in both groups (Table 1)

The overall exposure, estimated by the DAP, was similar for the two groups: 70,042 mGy cm^2^ in the Echosafe group and 75,207 mGy cm^2^ in the control group providing an adjustment ratio of 1.074. The mean DAP values were not statistically different between the two groups (*p* = 0.94), nor were the mean air kerma or time duration (fluoroscopy time). All data regarding the procedures are available in the appendix (Table S1 and S2).

### Radiation Measurement

The use of the Echosafe cabin resulted in a decrease in the cumulative radiation dose received by each body part studied, especially the right wrist, which tended to be heavily exposed with the control lead glass panel previously used in our institution (Figure 2, Table S3). Overall, the reduction in mean radiation dose was calculated to be between 56% and 78% when using the Echosafe cabin (mean of the 10 TLD dose measurements in each group).

### Ergonomic Assessment

**Figure 4:**
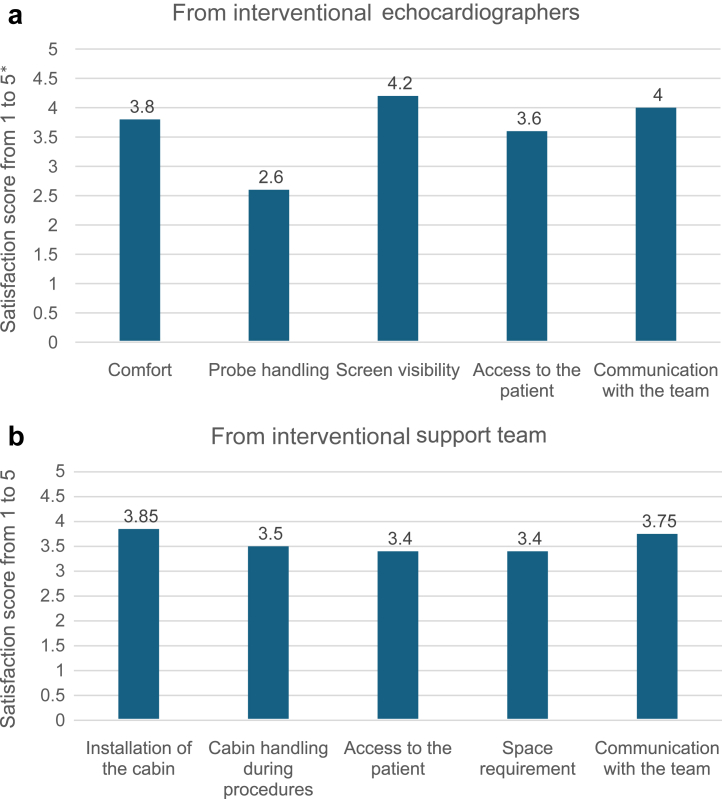
Overall satisfaction of the (a) interventional echocardiographers and (b) interventional support team with the Echosafe cabin. Six interventional echocardiographers and seven interventional support team members completed the satisfaction questionnaire. ∗A score of 1 was very bad and 5 was excellent.

We collected satisfaction questionnaires from six interventional echocardiographers and seven interventional support team members. This survey indicated that the echocardiographers considered the visibility through the screen and the communication with the patient or the team to be good (Figure 4). Postural comfort and patient accessibility were rated as fair to good, and transesophageal probe handling as bad to fair. The interventional support team assessed the cabin features as fair to good.

**Figure 5:**
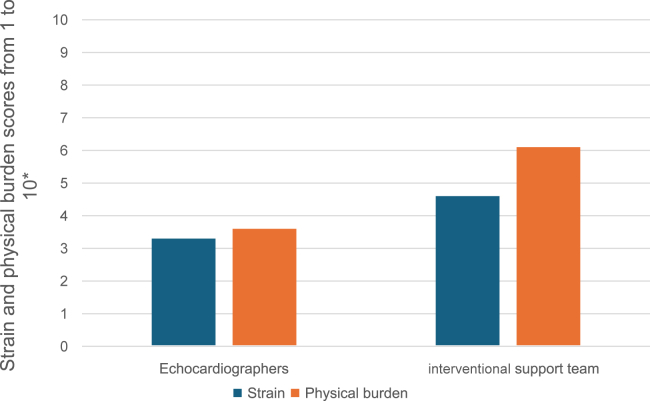
Evaluation of the strain and physical burden associated with the use of the cabin (from six interventional echocardiographers and seven interventional support team members). ∗A score of 1 corresponded to very light strain or physical burden and 10 was very high.

The strain and physical burden associated with the use of the cabin were rated as moderate by the echocardiographers and moderate to high by the interventional support team (Figure 5).

## Discussion

Our study supports the radioprotection efficiency of the Echosafe cabin with a numerically lower radiation exposure (by a factor of 2-5) compared to the device previously used in our hospital. With the cabin, the radiation dose measured on the shoulders, thorax, and left lens of the echocardiographer was below the detection limit (100 μSv). There was a noteworthy (76%) reduction in radiation on the right wrist, which remained, nevertheless, one of the most exposed areas. This reduction was likely due to the fact that, although the right wrist was in contact with the transesophageal probe, it was protected by the cabin lead flap, which had been designed for this purpose. However, decreasing the radiation that is related to the probe movements in the patient's mouth remains a challenge. The reduction in radiation on the left wrist was more modest (12%) than on the right wrist, but radiation in the control group was already low on the left wrist (170 μSv for all procedures), making further improvement difficult. In addition, the left hand was almost constantly on the ultrasound keyboard, slightly behind the cabin and outside the optimal protection field. Right lens radiation was reduced by 49%, which is substantial but still below the goal of complete radioprotection. This remaining radiation may be explained by the scattered radiation coming from the interventional cardiologists.

Our study found radiation levels (estimated by the DAP) identical to^10^ or lower^11^ than those described in the literature, which may be explained by the technological evolution of X-ray tubes or by the learning curve of the operators.

Regarding cabin ergonomics, the echocardiographers reported overall satisfaction, with good postural comfort, along with good screen visibility, patient accessibility, and team communication. Some discomfort related to limited mobility of the transesophageal probe was observed when echocardiography was performed in a standing position, although no such issues occurred when seated. This limitation required some adaptation for echocardiographers accustomed to working in a standing position. Nevertheless, no procedural failures were reported throughout the study. To improve ergonomics, further collaboration with Lemerpax has been performed to modify the cabin’s right lead flap, enabling the echocardiographer to work comfortably while standing. The interventional support team was satisfied with the cabin regarding patient accessibility and communication across the room but rated the physical burden as moderate to high (grade of 6.1/10) because of the weight and size of the cabin making the initial installation difficult.

This study has several limitations. First, as a preliminary study, we chose to use TLD detectors, which allow for reliable and multizone analysis of the echocardiographer’s exposure, but only provide a final cumulative dose of the radiation (corresponding to all procedures within each group). This study design is not suited to demonstrate statistical superiority and will require other studies with a different design. Second, we included a relatively small number of procedures, leading to heterogeneity in procedure duration and associated radiation. However, because the position of the echocardiographer remains unchanged during the procedure, we assume that the procedure type and duration do not influence the radiation protection performance of the cabin. Third, this is a single-center study and the Echosafe cabin was only compared with a lead glass panel, which was the radioprotective device used so far in our institution. It would be interesting to compare the Echosafe cabin with other devices, but to the best of our knowledge, there are no other radioprotective devices dedicated to the interventional echocardiographer. Fourth, certain factors that could have influenced radiation exposure—such as the distance between the table and the X-ray tube, angulation, and filtration—were not collected. Accounting for these variables in future studies would help to confirm the protective effectiveness of the cabin.

## Conclusion

The Echosafe cabin, dedicated to the radioprotection of the interventional echocardiographer, was associated with a numerically lower radiation exposure (by a factor of 2-5) compared to the device previously used in our hospital. The ergonomics and working comfort provided by the cabin are compatible with a daily use for transesophageal echocardiography-guided procedures in the catheterization laboratory, with moderate physical burden mostly related to the installation of the cabin. Further studies with larger sample sizes and different design will be needed to confirm the reduction in irradiation of the echocardiographer.

## Ethics Statement

The study was approved by the local ethics committee “Groupe Nantais d’Ethique dans le Domaine de la Santé”.

## Funding

The leaded glass door and the Echosafe cabin are the property of the department of cardiology of the Hospital.

## Disclosure Statement

Pierre-Guillaume Piriou has a consultancy agreement with Lemerpax.

The other authors had no conflicts to declare.
