# Supplementary material for: Performance of a Dedicated Radioprotection Cabin for the Interventional Echocardiographer During Structural Heart Procedures
Source: Struct Heart. 2025 Nov 10;10(3):100756. doi: 10.1016/j.shj.2025.100756 (PMC13000915; doi:10.1016/j.shj.2025.100756)
Supplement: Tables S1-S3 [file mmc1.docx]

*Table S1: population data and dosimetric indices for each procedure in the 'Echosafe' group.*

|  | Procedure | Age (year) | Sex (M/F) | Weight (Kg) | BMI (kg/m²) | Body surface (m²) | total DAP (cGy.cm²) | Air Kerma (mGy) | E (mSv) | Fluoroscopy time (min) |
| --- | --- | --- | --- | --- | --- | --- | --- | --- | --- | --- |
| 1 | Mitral- TEER | 89 | F | 68 | 25,9 | 1,779 | 857,8 | 59 | 2,40 | 16,93 |
| 2 | Mitral- TEER | 82 | H | 52 | 19,3 | 1,541 | 484 | 56 | 1,36 | 12,97 |
| 3 | Mitral- TEER | 79 | H | 68 | 21 | 1,837 | 6573,1 | 430 | 18,16 | 31,86 |
| 4 | Mitral- TEER | 77 | H | 78 | 25,8 | 1,959 | 2139,6 | 176 | 5,99 | 19,01 |
| 5 | Mitral valvuloplasty | 39 | H | 73 | 22 | 1,915 | 319,1 | 21 | 0,89 | 10,64 |
| 6 | Mitral paravalvular leak closure | 73 | F | 51 | 21 | 1,502 | 917,3 | 65 | 2,57 | 10,82 |
| 7 | Tricuspid-TEER | 56 | F | 58 | 20,8 | 1,646 | 575,7 | 41 | 1,61 | 16,22 |
| 8 | Mitral- TEER | 27 | F | 40 | 17,3 | 1,303 | 780,9 | 52 | 2,19 | 31,81 |
| 9 | Mitral- TEER | 77 | H | 92 | 27,8 | 2,172 | 2827,7 | 262 | 7,92 | 20,3 |
| 10 | Mitral- TEER | 79 | H | 96 | 32,8 | 2,182 | 3099,9 | 224 | 8,68 | 17,63 |
| 11 | Mitral- TEER | 73 | H | 74 | 22,8 | 1,923 | 3454,8 | 340 | 9,67 | 35,45 |
| 12 | Mitral paravalvular leak closure | 81 | F | 72 | 26,45 | 1,846 | 3776 | 417 | 10,57 | 44,28 |
| 13 | Mitral- TEER | 60 | H | 80 | 25,8 | 1,993 | 2880,2 | 324 | 8,06 | 31,47 |
| 14 | Mitral- TEER | 70 | H | 96 | 28,36 | 2,23 | 6849,8 | 593 | 19,18 | 34,79 |
| 15 | Mitral- TEER | 91 | H | 54 | 20,08 | 1,728 | 1318 | 12,117 | 3,69 | 19,12 |
| 16 | Mitral- TEER | 81 | H | 85 | 31,99 | 2,013 | 6454,1 | 521 | 18,07 | 44,88 |
| 17 | atrial septal defect occlusion | 30 | H | 85 | 25,38 | 2,084 | 286,8 | 21 | 0,83 | 4,18 |
| 18 | atrial septal defect occlusion | 57 | H | 72 | 22,22 | 1,895 | 137 | 10 | 0,35 | 2,82 |
| 19 | atriopulmonary fistula closure | 66 | F | 53 | 19,95 | 1,555 | 857,6 | 73 | 2,40 | 17,01 |
| 20 | Mitral paravalvular leak closure | 86 | F | 53 | 22,6 | 1,525 | 3617 | 386 | 10,13 | 16,97 |
| 21 | Mitral paravalvular leak closure | 86 | F | 53 | 21,8 | 1,534 | 1490,3 | 129 | 4,17 | 40,7 |
| 22 | Mitral- TEER | 74 | F | 91 | 41,5 | 2,03 | 2017,9 | 147 | 5,65 | 17,72 |
| 23 | Valve in valve TMVR | 78 | H | 89 | 31,5 | 2,083 | 6218,3 | 524 | 17,41 | 14,67 |
| 24 | atrial septal defect occlusion | 48 | H | 79 | 26,4 | 1,97 | 122,3 | 11 | 0,34 | 3,72 |
| 25 | atrial septal defect occlusion | 52 | H | 76 | 25,7 | 1,925 | 123,9 | 12 | 0,35 | 3,72 |
| 26 | atrial septal defect occlusion | 54 | H | 45 | 16,9 | 1,42 | 128,4 | 9 | 0,36 | 4,27 |
| 27 | atrial septal defect occlusion | 44 | F | 77 | 26,6 | 1,932 | 471,3 | 38 | 1,32 | 5,83 |
| 28 | atrial septal defect occlusion | 50 | H | 96 | 27,2 | 2,245 | 403,4 | 31 | 1,13 | 3,67 |
| 29 | Mitral- TEER | 61 | H | 58 | 24,5 | 1,606 | 1825,7 | 163 | 5,11 | 27,39 |
| 30 | Mitral- TEER | 82 | F | 52 | 22,2 | 1,51 | 1116,3 | 96 | 3,12 | 26,83 |
| 31 | Mitral- TEER | 90 | F | 48 | 21,9 | 1,43 | 1096 | 118 | 3,07 | 31,53 |
| 32 | atrial septal defect occlusion | 60 | F | 65 | 24,8 | 1,736 | 1144,9 | 86 | 3,21 | 9,85 |
| 33 | atrial septal defect occlusion | 49 | H | 80 | 25,8 | 1,993 | 341,9 | 22 | 0,96 | 3,9 |
| 34 | PFO closure | 44 | H | 90 | 26,87 | 2,15 | 240 | 24 | 0,67 | 2,53 |
| 35 | atrial septal defect occlusion | 17 | F | 63 | 21,3 | 1,737 | 782,1 | 53 | 2,15 | 1,83 |
| 36 | atrial septal defect occlusion | 30 | F | 56 | 21,3 | 1,60 | 91,2 | 7 | 0 | 0,05 |
| 37 | atrial septal defect occlusion | 34 | F | 65 | 26,4 | 1,72 | 213,8 | 13 | 0,60 | 5,38 |
| 38 | atrial septal defect occlusion | 60 | H | 82 | 26,4 | 2,02 | 260,5 | 26 | 0,73 | 3,53 |
| 39 | Mitral- TEER | 87 | H | 73 | 26,8 | 1,86 | 2022,8 | 140 | 5,66 | 15,17 |
|  |  |  |  |  |  |  |  |  |  |  |
| 40 | Valve in ring TTVR | 56 | F | 84 | 32,8 | 1,99 | 1724,6 | 148 | 4,83 | 6,42 |

*population data and dosimetric indices for each procedure in the 'Echosafe' group".*

*PFO: patent foramen ovale; TEER: transcatheter edge to edge repair ; TMVR: transcatheter mitral valve replacement ; TTVR: Transcatheter Tricuspid Valve Replacement*

*Table S2 - population data and dosimetric indices for each procedure in the control group.*

| N° procédure | Type de procédure | Age (year) | Sex (M/F) | Weight (Kg) | BMI (kg/m²) | Body surface (m²) | total DAP (cGy.cm²) | Air Kerma (mGy) | E (mSv) | Fluoroscopy time (min) |
| --- | --- | --- | --- | --- | --- | --- | --- | --- | --- | --- |
| 1 | Mitral- TEER | 88 | H | 62 | 21,5 | 1,716 | 3469,3 | 245 | 9,71 | 44,67 |
| 2 | Mitral- TEER | 87 | H | 70 | 24,8 | 1,828 | 1987,2 | 148 | 5,56 | 23,49 |
| 3 | Mitral paravalvular leak closure | 81 | F | 77 | 28,3 | 1,915 | 12908,6 | 1748 | 36,15 | 51,97 |
| 4 | Mitral- TEER | 66 | H | 123 | 35,2 | 2,562 | 26773,2 | 2252 | 74,96 | 67,3 |
| 5 | Mitral- TEER | 89 | F | 60 | 23,4 | 1,655 | 2129 | 166,84 | 5,96 | 35,74 |
| 6 | Mitral- TEER | 69 | F | 68 | 25 | 1,789 | 485,6 | 41 | 1,36 | 6,36 |
| 7 | atrial septal defect occlusion | 64 | H | 102 | 33,3 | 2,27 | 952,1 | 67 | 2,67 | 3 ,3 |
| 8 | atrial septal defect occlusion | 23 | F | 51 | 21,2 | 1,499 | 207,6 | 14 | 0,58 | 10,11 |
| 9 | atrial septal defect occlusion | 61 | F | 58 | 22,7 | 1,625 | 131,6 | 8 | 0,37 | 3,75 |
| 10 | Mitral- TEER | 83 | H | 70 | 22,8 | 1,850 | 7646,8 | 532 | 21,41 | 48,84 |
| 11 | Mitral- TEER | 72 | H | 78 | 30,1 | 1,914 | 3775,9 | 306 | 10,57 | 19,67 |
| 12 | Mitral- TEER | 93 | H | 53 | 22,1 | 1,531 | 1402,5 | 108 | 3,93 | 19,11 |
| 13 | Mitral paravalvular leak closure | 81 | F | 72 | 26,4 | 1,846 | 5013,4 | 614 | 14,04 | 63,64 |
| 14 | Mitral- TEER | 91 | F | 63 | 22,3 | 1,725 | 2488,9 | 254 | 6,97 | 20,3 |
| 15 | Mitral- TEER | 74 | H | 63 | 22,1 | 1,728 | 2326,8 | 230 | 6,52 | 28,7 |
| 16 | atrial septal defect occlusion | 32 | H | 66 | 21,55 | 1,792 | 666,4 | 60 | 1,86 | 9,93 |
| 17 | atrial septal defect occlusion | 25 | F | 55 | 22,35 | 1,569 | 137,6 | 11 | 0,39 | 4,42 |
| 18 | atrial septal defect occlusion | 24 | F | 61 | 22 | 1,692 | 125,6 | 11 | 0,35 | 4,63 |
| 19 | atrial septal defect occlusion | 61 | F | 80 | 30,11 | 1,948 | 104,2 | 9 | 0,29 | 2,67 |
| 20 | Mitral- TEER | 88 | H | 64 | 22,14 | 1,746 | 1182,5 | 87 | 3,31 | 16,88 |
| 21 | Mitral- TEER | 86 | F | 70 | 27,3 | 1,801 | 775,8 | 56 | 2,17 | 11,61 |
| 22 | Mitral- TEER | 84 | F | 54 | 21,6 | 1,556 | 353 | 26 | 0,99 | 14,92 |
| 23 | atrial septal defect occlusion | 48 | H | 73 | 27,1 | 1,857 | 163,3 | 11 | 0,46 | 2,71 |
| 24 | atrial septal defect occlusion | 25 | F | 52 | 21.1 | 1.521 | 77.5 | 5 | 0.22 | 3,07 |

*TEER: transcatheter edge to edge repair*

Table S3: Radiation measurements (expressed in µSv by the thermoluminescent dosimeter) for each body part in each group, along with the percentage of radiation reduction in the Echosafe group compared to the control group (after adjustment for a factor of 1.074 to account for the DAP difference).

| **Thermoluminescent dosimeter** | **Control group**  **(µSv)** | **Echosafe group**  **(µSv)** | **Radiation reduction (%)**  *(after adjustment by the DAP difference between the two groups)* |
| --- | --- | --- | --- |
| Right lens | 380 | 180 | 49% |
| Left Lens | 110 | <100 | 19-100% |
| Right Shoulder | 170 | <100 | 48-100% |
| Left Shoulder | 230 | <100 | 61-100% |
| Thorax | 410 | <100 | 79-100% |
| Right wrist | 1230 | 280 | 76% |
| Left wrist | 170 | 140 | 12% |
| Pubis | 230 | 110 | 49% |
| Right ankle | 710 | 450 | 32% |
| Neck | <100 | <100 | NA |
